# Editorial: Glycans as Players in Host-Microbe Interactions: A Structural Perspective

**DOI:** 10.3389/fmolb.2021.791765

**Published:** 2021-11-03

**Authors:** James A. Garnett, Angelina Sá Palma, Bing Liu, Yan Liu

**Affiliations:** ^1^ Centre for Host-Microbiome Interactions, Faculty of Dental Oral and Craniofacial Sciences, King’s College London, London, United Kingdom; ^2^ UCIBIO, Applied Molecular Biosciences Unit, Department of Chemistry, School of Science and Technology, NOVA University Lisbon, Caparica, Portugal; ^3^ Associate Laboratory i4HB—Institute for Health and Bioeconomy, School of Science and Technology, NOVA University Lisbon, Caparica, Portugal; ^4^ Institute for Protein Science and Phage Research, The First Affiliated Hospital of Xi’an Jiaotong University, Shaanxi, China; ^5^ Glycosciences Laboratory, Department of Metabolism, Digestion and Reproduction, Imperial College London, London, United Kingdom

**Keywords:** glycans and glycoconjugates, glycosidic linkage, microbe, cell surface, host interaction

Glycans have highly complex and dynamic structures. Small variations in their composition and types of linkage may lead to significant changes in their biochemical and biophysical properties and are determinant for their specific recognition by proteins. Microbial recognition of glycans represents major biological processes including adhesion, biofilm formation, host invasion, and signalling. In some diseases, alterations in the ability of microbial pathogens to bind glycans can lead to dysbiosis and result in inflammation. On the other hand, microbial glycans are recognized as key mediators of the host immune response, although the presentation of host-like glycans on microbial surfaces can be used to evade this detection. To advance our understanding of these complex interactions at the atomic level, structural-based studies and in the development of new techniques are key.

In this Research Topic of *Frontiers of Molecular Biosciences*, we have eight articles taking us to the frontiers of knowledge on the roles of glycans in mediating host-microbe interactions during infection and in stimulating a host immune response, from a structural perspective. These emphasise the importance of glycans during host-microbial invasion/colonization, host responses to invading pathogens, microbial subversion of host responses and the development of new vaccines.

Siukstaite, Imberty and Romer review the structural diversity in lectins that bind the glycosphingolipid globotriaosylceramide (Gb3) and their different binding mechanisms (Siukstaite et al.). Glycolipids are common cell surface structures and Gb3 is a host target receptor for a range of microbial pathogens. As well as delivering an overview, the authors illustrate in detail the structural basis for Gb3-binding by three bacterial lectins: Shiga toxin B-subunit (StxB) binding to Gb3 and inducing clathrin-dependent endocytosis and toxin uptake; Gb3 recognition by the *Pseudomonas aeruginosa* lectin LecA and subsequent bacterial membrane engulfment; and host recognition by the *Streptococcus suis* SadP and uropathogenic *Escherichia coli* PapG adhesins. The review also covers structural features of Gb3-binding lectins from fungi, plants and animal sources and discuss the evolution for recognition of this glycan epitope.

Staying focussed on bacterial systems, Matos and Reis describe the role of host glycans during colonization of the human gastric mucosa by *Helicobacter* species (Matos et al.). They focus on three *Helicobacter pylori* adhesins: the blood-group antigen binding adhesin (BabA), the sialic acid-binding adhesin (SabA), the LacdiNAc-binding adhesin (LabA), and discuss their roles in *H. pylori* colonization of human gastric mucosa. In addition, they describe the knowledge so far on the non-*H. pylori Heliobacter* (NHPH) species which lack the above-mentioned *H. pylori* adhesins. The adhesion molecules used by NHPH and the full host adhesion mechanisms remain to be elucidated.

The next group of articles in this Topic address the role of glycans in host-viral interactions. Sasisekharan and colleagues provide a structural perspective of the interactions between host or viral glycoproteins with their complementary viral or host glycan binding proteins (Miller et al.). Using a structure-based rationale of the recognition by lectins and antibodies they categorize “glycoepitopes” on the virus surface glycans in three classes: minimal glycoepitopes, glycan-protein glycoepitopes and topological glycoepitopes. They further discuss the potential value of topological glycoepitopes in the development of novel antiviral targets, using as example the recognition of HIV envelope glycoprotein by the neutralizing 2G12 antibody.

The next article by Duan and colleagues concentrates on Rotavirus, a highly infectious viral pathogen that leads to acute gastroenteritis in children (Sun et al.). Here the authors review the recent advances in the structural basis for host glycan recognition by VP8*, the distal region of the viral outer capsid spike protein and the link with host tropism. The final article of this group (Zhao et al.) is highly topical and reviews the glycosylation of spike protein and other proteins in SARS-CoV-2, highlighting their role in mediating host interactions and helping to evade host immune responses. They also discuss the potential role of glycans for increasing the protective immune response of spike protein-based vaccines.

Further into the development of vaccines, the inclusion and optimization of vaccine adjuvants are essential to produce a stronger immune response to the antigen. This is tackled by Liu and Zhao in their review (Wang et al.) in which polysaccharides from traditional Chinese medicines (or natural products) and their role in modulating human immune responses are presented and the potential of these polysaccharides as vaccine adjuvants are discussed.

Mammalian lectin receptors have a major role in recognizing glycans presented on the surface of invading pathogens and this is reviewed by Yamaguchi and colleague (Manabe and Yamaguchi et al.). Lectin co-operation, oligomerization and multiple site binding is discussed and how this allows specificity in the detection microbial glycans.

In the final article of this Topic (Pitangui et al.), Paracoccin (PCN) from the human fungal pathogen *Paracoccidioides brasiliensis* is overviewed. This protein is an archetypal example of a glycan binding protein with multiple functions. The authors review its role as a chitinase but also its interactions with host Toll-like receptors and subsequent stimulation of protective immunity.

We hope that with this Research Topic the reader will find a useful selection of reviews that brings together the importance of glycans in supporting host-pathogen interactions and stimulates new ideas for using glycans to target and develop new therapies.

